# The Compound Expression of HSP90 and INOS in the Testis of Diabetic Rats as Cellular and Pathologic Adverse Effects of Diabetes

**DOI:** 10.1155/2020/3906583

**Published:** 2020-06-27

**Authors:** Ali Alsarhan, Kawther Faisal Amawi, Inas Saleh Al-Mazari, Hashem Abu Hurirah, Ahed J. Alkhatib

**Affiliations:** ^1^Department of Pharmaceutical Sciences, Faculty of Pharmacy, Jadara University, Irbid, Jordan; ^2^Faculty of Pharmacy, Zarqa University, Jordan; ^3^Department of Legal Medicine, Toxicology of Forensic Science and Toxicology, School of Medicine, Jordan University of Science and Technology, Jordan

## Abstract

**Introduction:**

Diabetes is increasingly prevalent at global level and associated with various impacts including the male reproductive system.

**Aims:**

This research is aimed at investigating the influence of diabetes on the localization and expression of HSP90 and iNOS in the testicular tissue of diabetic rats.

**Methods:**

A diabetic model was developed through a single injection of alloxan monohydrate intraperitoneally (purchased from Sigma-Aldrich) 120 mg/kg body weight following fasting for 12 hrs. The experiment involved two groups, the control and diabetic groups with 10 albino rats in each group. Diabetes was considered if glucose concentration was ≥200 mg/dl. The experiment duration was for one month. After the experiment had finished, all rats were terminated and prepared for routine histological and immunohistochemical examination.

**Results:**

The results revealed that diabetes caused morphological changes at histological level in testicular tissue. Immunohistochemical examination showed that diabetes significantly upregulated the expression of both HSP90 and iNOS in the testicular tissue of diabetic rats as compared with that of the control group (*p* < 0.001).

**Conclusion:**

Diabetes may induce adverse health effects on the male reproduction through upregulation of HSP90 and iNOS in the testicular tissue of diabetic rats.

## 1. Introduction

Diabetes is not a disease, but it is rather a sum of diseases known as metabolic diseases resulting from increased glucose levels (hyperglycemia) due to insulin insufficiency as in terms of either secretion or function or both [1, 2].

Long-term hyperglycemia is associated with systemic defects including vision, renal system, peripheral nerves, and cardiovascular system [2, 3].

There is a loss of insulin secretion resulting in insulin deficiency associated with type 1 diabetes [4]. The autoimmune attack by T-cells to pancreatic beta cells is believed to be the reason beyond type 1 diabetes [4, 5].

Autoimmunity is thought to cause type 1 diabetes that leads to insufficient production of insulin by pancreatic beta cells [4, 6]. Insulin insufficiency impacts the whole metabolic activities of the body [7]. There are various complications associated with bad management of type 1 diabetes such as cardiovascular diseases, diabetic neuropathy, and diabetic retinopathy [8–12]. Type 1 diabetic patients who are under well management can suffer from damage in peripheral organs related to diabetic complications [13, 14].

Diabetes causes troubles in various organs such as the reproductive system in several species at both sexes [15–17].

There are adverse effects of type 1 diabetes against the testicular function on cellular level and the production of testosterone [18]. However, among patients with diabetes, the development of oxidative stress has been shown to deteriorate the testicular function [19].

The main function of the testis is to produce testosterone and sperm. These functions work to retain the characteristics of male and species preservation [20]. The testis of mammals is lined by a capsule composed of a fibrous tissue known as the tunica albuginea for protective purposes [21]. Reactive oxygen species (ROS) disturbs performance of the testicular functions [22, 23].

Heat shock protein (HSP) 90 exists in cells as a chaperone that acts in controlling cellular changes such as protein refolding, removing denatured proteins through the process of the proteasome [24]. HSP90 participates in making what is called the machine of chaperone [24]. Various types of stress can induce the production of HSP90 including cancer and diabetes [24]. Several studies pointed that the overexpression of HSP90 has negative impacts such as lowering cytoprotective induction of other HSPs including HSP40 and HSP70 through interaction with heat shock factor 1 (HSF-1) [25–28].

Nitric oxide (NO) is known by its ability to induce signals in several tissues and works to control a variety of physiological and cellular processes. Overproduction of NO may occur in some clinical conditions including the cardiovascular system as well as diabetes that impacts the vascular functions [29, 30].

### 1.1. Study Aims

The main aim of his study was to explore both the localization and expression of HSP90 and iNOS in the testicular tissue of diabetic rats.

## 2. Methods and Materials

### 2.1. Preparation of Animals and Diabetic Model

Twenty rats (males, albino) were selected in randomization approaches and subdivided to either the control group (*N* = 10) or the diabetic group (*N* = 10). We conducted this study at the Department of Biology that is hosting animal house unit in Yarmouk University, Jordan. According to the instructions of the university, IRB gave the approval of this study. Animals were bought from the animal unit. Animals were weighted before the initiation of the experiment. The mean of animal's weight was 185 ± 7.3 grams. A private room in the animal house was hosted for this study in which animals were placed in cages. One week prior to the conduction of research, all rats were treated as the same and were exposed to the same environmental conditions for acclimatization purposes.

Diabetes in rats was developed in rats using one dose of alloxan monohydrate (Sigma-Aldrich) through intraperitoneal injection (120 mg/kg) following 12-hour fasting. Blood glucose was measured daily to assure that animals were hyperglycemic (≥200 mg/dl) using a commercial device (Glucocheck, HomeMed (Pty) Ltd.). All animals were terminated at the end of the study (study duration was 1 month). The testes were removed, washed in normal saline, and fixed in 10% formalin for 24 hrs; then they were processed and stained for hematoxylin and eosin for routine histological examination, and other sets were stained for immunohistochemistry to examine the localization, expression, and immunoreactivity of HSP90 and iNOS. We have developed our protocols for immunohistochemistry in our laboratory and previously published them [31–33].

### 2.2. Immunohistochemistry Protocols

Testis tissue samples were processed, sectioned, and mounted on charged slides. Sections were then passed from deparaffinization till water. Immunohistochemistry staining protocols started by placing sections in a container containing a solution of 1% hydrogen peroxide for 20 minutes to neutralize internal cellular activity of peroxidase enzyme. A washing step by phosphate-buffered saline (PBS) (pH = 7.2-7.4) was followed, and then 1% bovine serum albumin (BSA) was used to overcome nonspecific binding. After washing with PBS, primary monoclonal antibody solution (iNOS, 1 : 100/HSP90, 1 : 100; Santa Cruz Biotechnology) was incubated with sections for 1 hr using a humid chamber. A washing step by PBS was performed, and then incubation with secondary biotinylated antibodies for 20 minutes was followed, then washed with PBS and incubated with streptavidin conjugated with horseradish peroxidase enzyme for 20 minutes, followed by washing with PBS carried out. Immunohistochemical reactions were viewed through the reaction with DAB (diaminobenzidine) until the reaction had developed (brown color), and the reaction was finished by washing with tap water. Hematoxylin was used as a counterstain for 30 seconds; then sections were dehydrated and mounted with mounting medium.

### 2.3. Explanation of the Results

We evaluated the expression of HSP90 and iNOS based on the reading of output of Adobe Photoshop Software version 7.2. Micrographs representing immunostained sections were interpreted in terms of pixels. The pixels represented the colors of the biomarker (brown) and the color of the remaining tissue (blue). The number of pixels representing the color of the biomarker is divided by the whole number of pixels (the sum of brown and blue) to give a ratio of expression.

### 2.4. Statistical Analysis

We used the software SPSS version 21.0 to analyze the data. Differences in the means were computed using an independent *t*-test. Significance was considered if *α* < 0.05. Data representing iNOS and HSP90 for each group were used as the mean ± standard deviation.

## 3. Results

### 3.1. Biochemical Findings of Study

Table 1 shows that the mean of glucose in the control group was 107.8 ± 8.6 mg/dl, while that in the diabetic group was 297.4 ± 22.5 mg/dl. The difference in means was statistically significant (*p* = 0.001). The mean of insulin was 2.25 ± 0.22 ng/ml in the control group, and in the diabetic group, the insulin mean was 0.87 ± 0.12 ng/ml. The difference in means was statistically significant (*p* = 0.001).

### 3.2. Histological Changes of Rat Testicular Tissue in the Control and Diabetic Groups

**Figure 1:**
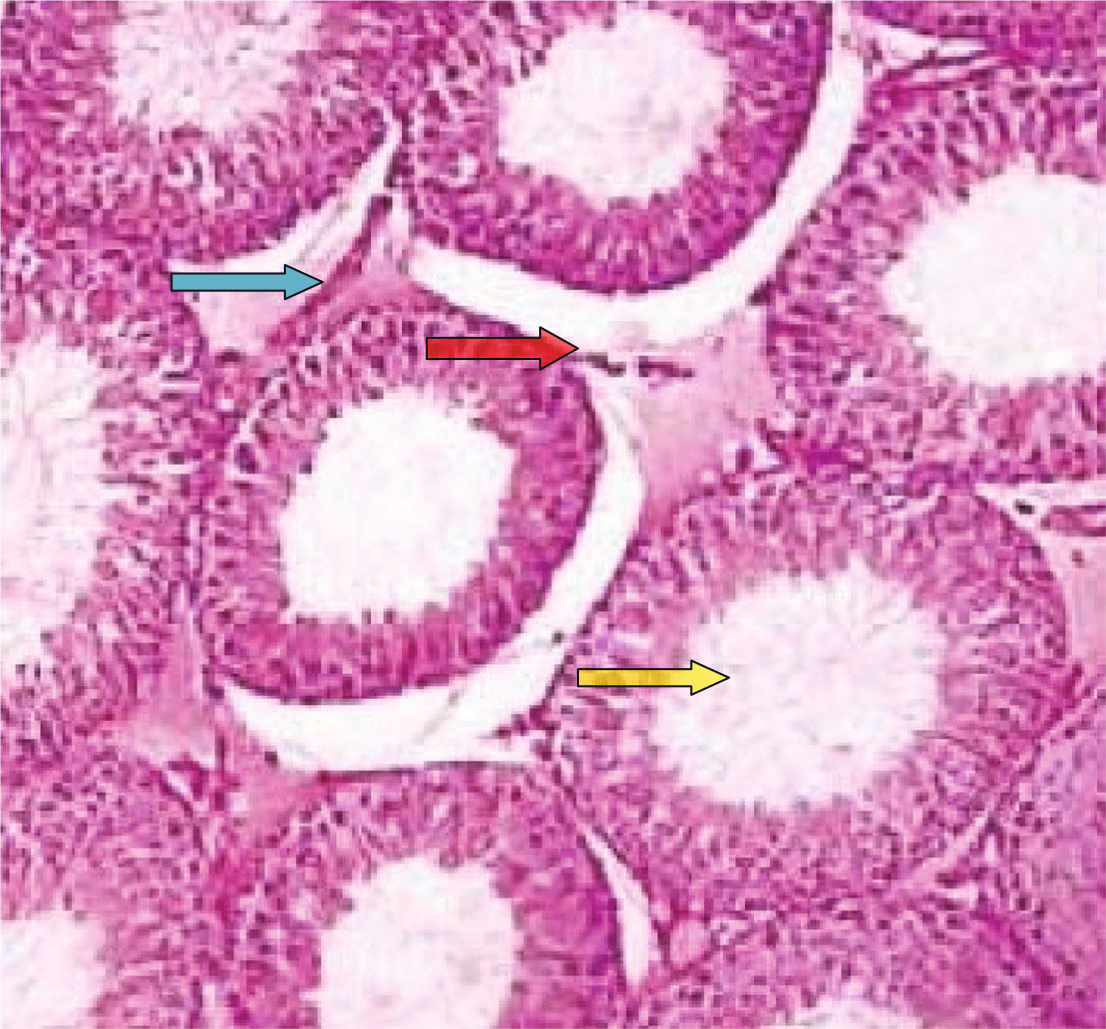
Histology of testicular tissue in the control group. A blood vessel is indicated by blue arrow, Leydig cells (red arrow), and seminiferous tubules (yellow arrow). H and E stain (40x magnification).

As seen in Figure 1, normal histology of rat testicular tissue is presented. There are a blood vessel (green arrow), Leydig cells (red arrow), and seminiferous tubules (yellow arrow).

**Figure 2:**
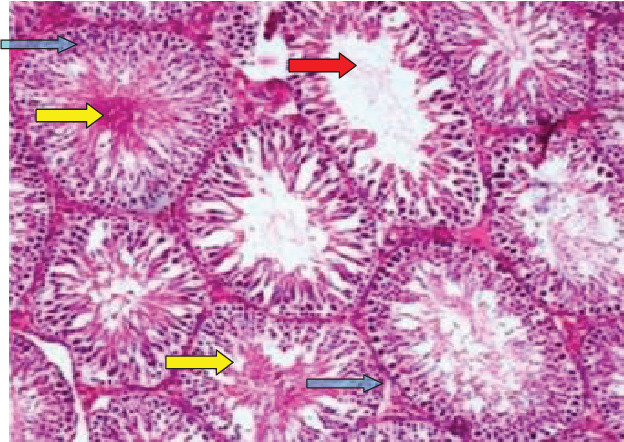
Histological changes of diabetic testicular tissue. Three features are seen: (a) inflammatory reactions observed as increased inflammatory cells (blue arrows), (b) no well-preserved architecture of seminiferous tubules (red arrow), and (c) congestion of seminiferous tubules (yellow arrow). H and E stain (40x magnification).

As demonstrated in Figure 2, diabetes induced some histological changes in the testicular tissue such as the existence of inflammatory cells (blue arrows), congestion (yellow arrows), and transforming changes in seminiferous tubules from being oval to being elongated (red arrow).

### 3.3. Immunohistochemical Studies

We studied the localization and expression of HSP90 and iNOS in the study groups.

#### 3.3.1. The Level of HSP90 in Study Groups

As seen in (Table 2), the level of HSP90 in the control group was 0.17 ± 0.035, and diabetes significantly increased the level of HSP90 (0.27 ± 0.025) (*p* < 0.001).

#### 3.3.2. The Immunohistochemical Expression of HSP90 in Study Groups

**Figure 3:**
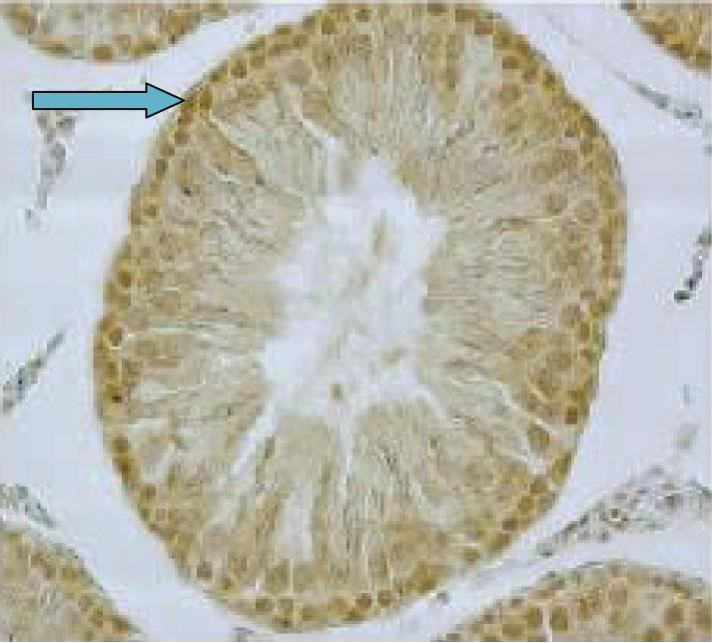
The expression of HSP90 in the control group. Nuclear stain is indicated in the nucleus of seminiferous tubules (blue arrow) (40x magnification).

**Figure 4:**
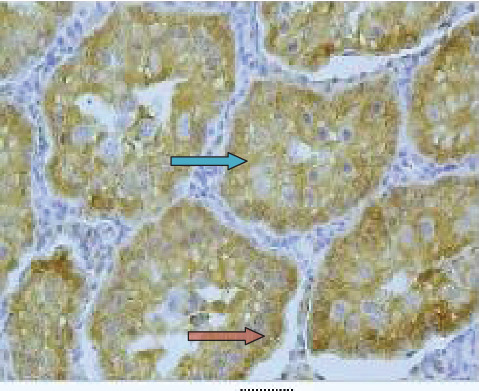
The localization and expression of HSP90 in the testicular tissue of diabetic rats. The arrows point to expression of HSP90 in the cytoplasm (blue arrow) and nucleus of cells (red arrow) in seminiferous tubules (40x magnification).

As demonstrated in Figure 3, the expression of HSP90 was located in the nucleus as indicated by the arrow in seminiferous tubules. Diabetes led to significantly increased expression of HSP90 in testicular tissue and seminiferous tubules (Figure 4) as compared to the control group (*p* < 0.001). Moreover, the expression of HSP90 is obvious in both the cytoplasm and nucleus due to the diabetic effects.

#### 3.3.3. The Level of iNOS in the Control and Diabetic Groups

As indicated in (Table 3), the mean level of iNOS in the testicular tissue in the control group was 0.16 ± 0.032, and that was significantly less than that in the diabetic group (0.25 ± 0.031) (*p* < 0.001).

#### 3.3.4. The Immunohistochemical Expression of iNOS in Study Groups

**Figure 5:**
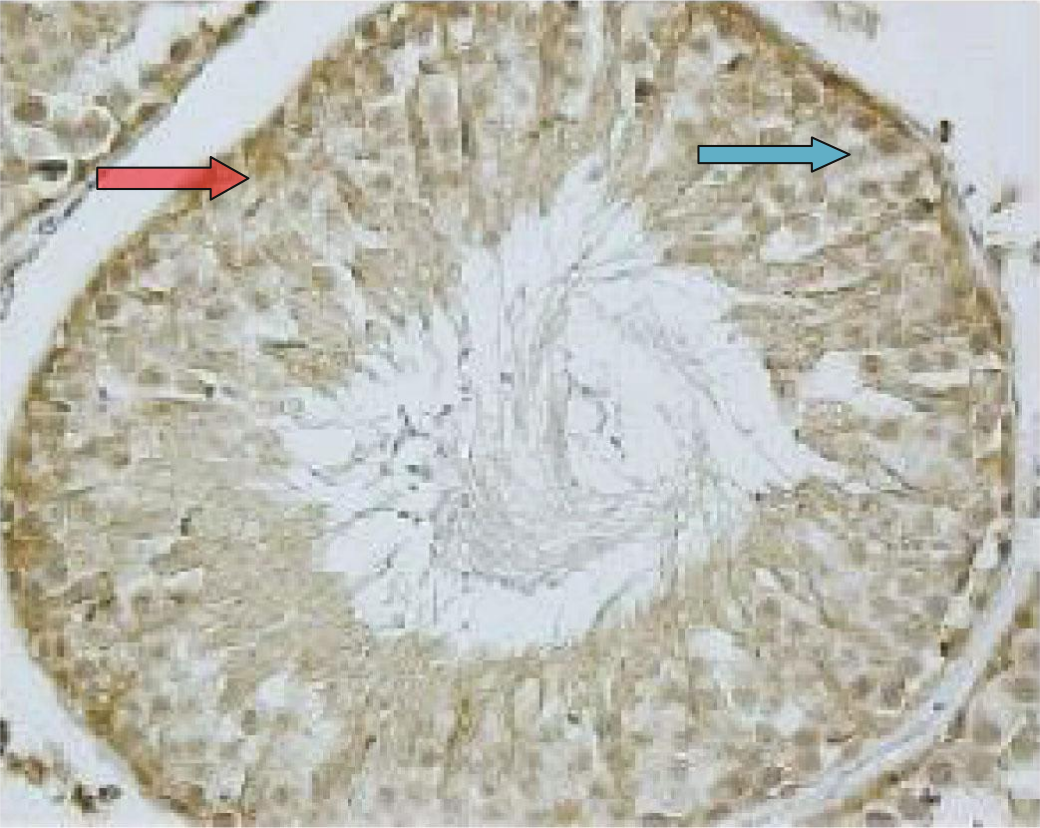
The expression and localization of iNOS in the testicular tissue in the control group. The arrows point cytoplasmic reactivity (red arrow) and nuclear localization of iNOS (blue arrow) in seminiferous tubules (40x magnification).

As seen in Figure 5, the expression of iNOS was not strong in terms of intensity and was localized mainly in the nucleus (blue arrow) and to a less extent in the cytoplasm of seminiferous tubules (red arrow).

**Figure 6:**
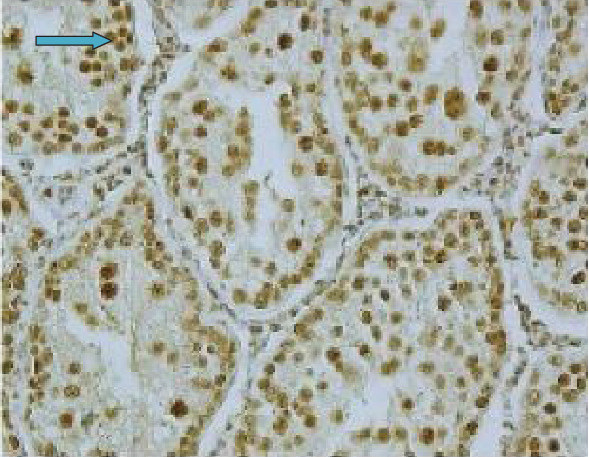
The expression and localization of iNOS in the testicular tissue of diabetic rats. Strong expression of iNOS is observed in the diabetic group as compared with control group (Figure 5). Arrow (blue) points to nuclear localization of iNOS (40x magnification).

As indicated in Figure 6, diabetes increased the expression of iNOS seminiferous tubules of testicular tissue. The localization of iNOS was prominent in the nucleus (blue arrow).

## 4. Discussion

The results of the present study showed that the diabetic model was successfully induced as reflected by both the glucose level (297.4 ± 22.5 mg/dl) and the insulin level (0.87 ± 0.12 ng/ml). As compared to control, the differences were statistically significant. These findings showed that the induction of the diabetic model permits the development of further changes in histological and immunohistological levels. These findings confirmed previous studies that were based on the induction of diabetes [1, 2, 4, 5]. On the histological level, the results of this study showed different types of changes including the development of inflammatory changes as reflected by eosinophilic changes, the lack of well preservation of seminiferous tubules, and the lack of well intact between seminiferous tubules. These changes are thought to affect the main functions of the testicular tissue such as the production of testosterone and sperms [20, 34–36].

The results of the present study showed that HSP90 was significantly expressed in the testicular tissue of diabetic rats compared with control group rats (*p* < 0.001), although HSP90 is potentially important in offering cytoprotection at physiological limits [24]. But in the case where its upregulation is increased, it is expected to develop negative impacts through inhibition of production of other HSPs that lead to the cytoprotection [25–28].

The results of the present study showed that iNOS was significantly expressed in the testicular tissue of diabetic rats compared with the testicular tissue in rats of the control group. This implies that the testicular tissue of diabetic rats is impacted by events of the oxidation process that leads to the infertility problems. Such trends were discussed in other studies [37–40].

This study showed synergistic effects of both HSP90 and iNOS impacting the testicular tissue of diabetic rats. Appropriate therapeutic strategies for diabetes and infertility problems resulting from diabetes may be achieved through lowering the expression of HSP90 and iNOS.

## 5. Conclusion

Diabetes type 1 impacts testicular functions through synergistic actions of HSP90 and iNOS.

## Figures and Tables

**Table 1:** The mean levels of glucose and insulin in the study groups.

Variable	Control group		Diabetic group		*p* value
	M	SD	M	SD	
Glucose (mg/dl)	107.8	8.6	297.4	22.5	0.001
Insulin (ng/ml)	2.25	0.22	0.87	0.12	0.001

**Table 2:** The level of HSP90 in the study groups.

Variable	Mean	SD	Significance
HSP90:			<0.001
Control	0.17	0.035	
Diabetic	0.27	0.025	

**Table 3:** The level of iNOS in the study groups.

Variable	Mean	SD	Significance
iNOS:			*p* < 0.001
Control	0.16	0.032	
Diabetic	0.25	0.031	

## Data Availability

The experimental data used to support the findings of this study are included within the article.
